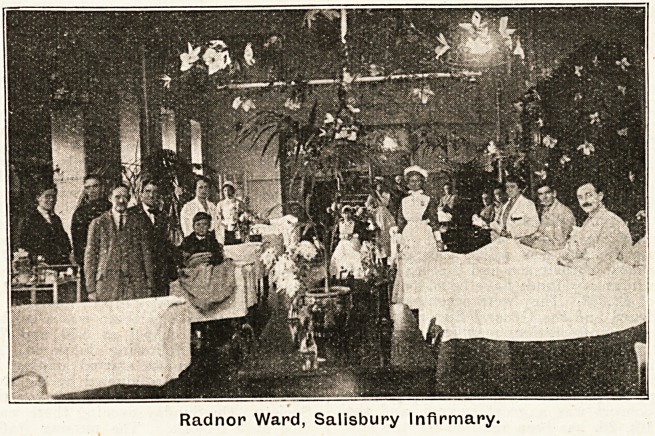# Christmas in the Hospitals

**Published:** 1920-01-10

**Authors:** 


### CHRISTMAS IN THE HOSPITALS.

### DREADNOUGHT HOSPITAL, GREENWICH.

CHRISTMAS was celebrated at the Seamen's Hospital in a manner befitting the best traditions of this festive season. Great ingenuity and artistic talent had been displayed by the sisters and by some of their convalescent patients in transforming their floors into pleasant harbours of greenery lit by fairy lamps and shaded electric lights, which lent a Christmas pantomime atmosphere to all the proceedings of the day. In one corridor a model lighthouse lashed by a card-board sea, flashed its intermittent light to a passing ship; in another the festoons of paper wisteria and the Oriental lampshades must have brought thoughts of home to the minds of the Japanese patients.

On Christmas Eve the choir of St. Alfege's Church spent two hours in the hospital, visiting each floor, and their delightful singing of Christmas hymns and carols was a pure joy to listen to. On Christmas Day services were held in the chapel by the Chaplain. At 12.30, the patients who were able to leave their beds sat down at well-loaded tables and paid the best compliment possible to those who had provided the fare by consuming it with wholehearted relish. In addition to the turkeys and plum pudding there was an ample supply of fruit, biscuits, sweets, and tobacco which had been received from many kind supporters of the hospital. In the evening an entertainment was given by the "Checks" Concert Party, whose breezy repertoire was received with unadulterated pleasure by a wholly appreciative audience.

### EAST LONDON HOSPITAL FOR CHILDREN.

CHRISTMAS in the wards of the East London Hospital for Children presented the same joyous scenes as its predecessors. Each ward was becomingly decorated, many of the decorations having been made by the children themselves. The children hung their stockings up overnight, and Santa Claus did not forget to visit each cot, and was as liberal as usual with his gifts. There was a Christmas tree in each ward, and much feasting.

The usual Christmas party, which has been in abeyance during the war, was held on Thursday, January 1. The hospital and wards were gaily decorated, and the many visitors enjoyed themselves for the first hour visiting the little occupants. Then a move was made to the out-patients' department, and many willing hands conveyed the little sufferers to the large out-patient hall, where further delights in the shape of marionettes and a Guards' band filled up another pleasant hour. Then at a given signal two monster trees laden with gifts were lit up, and the real fun of the afternoon began. Father Christmas appeared out of an enormous cracker, and distributed the many gifts with his usual lavishness. That night many tired but happy little faces could be seen fast asleep, and hugging as they slept dolls, Teddy Bears, and other priceless possessions. Toys were received from H.M. the Queen, Lady Beatty, Lady Haig, and other well-known people.

### GREAT ORMOND STREET.

A HAPPY throng of 250 children—looking almost too well to be there at all—crowded the big out-patients' hall at the Hospital for Sick Children on the Saturday in Christmas week, and cheered themselves hoarse at the arrival of Santa Claus (at other times Mr. White, the casualty officer). Excitement after excitement—in the forms of a concert, a tea, and a cinematograph entertainment—culminated in the joyous dismantling of that prodigy of Christmas-trees that reared its glittering height in one corner of the room.

On December 30 it was the turn of the little in-patients, whose pleasure had already been half fulfilled in glorious anticipation as they peeped from their cots at the wondrous preparations being made on every side.

### HAMPSTEAD GENERAL HOSPITAL.

CHRISTMAS at the Hampstead General Hospital was not less happy than it usually is, but many missed the sprinkling of "blue" and the jovial spirits that not the wounds of battle could suppress, for all the soldiers left last spring. Father Christmas was as bountiful as ever, and did not limit his attentions to the children, whose stockings he well filled. The hospital was awakened by Christmas carols sung by the nurses, and none could wish, in times of sickness, to awaken in surroundings more pleasant than the delightful wards, for which the hospital is noted, beautified by decorations upon which much artistic talent had been bestowed. Dinners were served at noon, when members of the visiting staff enjoyed the privilege of carving the turkeys and hams and dividing the puddings, which were none the worse for having been well stirred before boiling. As Christmas Day fell on visiting day the patients had their usual number of visitors, but each patient was allowed to invite one to tea. The chief feature of the season was the children's party, when many little ones, present and past patients, were entertained and participated in the distribution from the magnificent Christmas tree. A happy Christmas it was undoubtedly, and though not boisterous, on the other hand there has been no unpleasant reaction.

### BEDFORD COUNTY HOSPITAL.

THE best Christmas on record is the unanimous report, only spoilt by the illness of our matron. Proceedings commenced with carols sung on Christmas morning by the sisters and nurses. Sumptuous meals, including three helpings of turkey at dinner, were popular with the patients, and our good secretary, Mr. B. Wadmore, made a delightful Father Christmas, handing out suitable gifts to all, with a few appropriate words. Mr. Panis, our organist, kindly took a party of songsters round the wards in the afternoon of Christmas Day. On Boxing Day the nurses' dinner, followed by a dance and games, went with a delightful swing, and was followed on Saturday by a successful party for the domestic staff.

Three ward concerts were held during the following week, in which the day nurses gave a delightful "gipsies'" entertainment, the dresses and singing being equally good. The night nurses produced a wonderful Jazz-band entertainment, and the sisters were very much to the fore with a grand performance of "Mrs. Jarley's Waxworks." Mr. Panchand, our radiographer, was a host in himself with his humorous songs, and brought along the "Doughnuts" party on one evening.

The whole of the staff throughout the festive season worked with that whole-hearted sympathy which always characterises them and makes the County Hospital the happy home it is.

### THE GENERAL INFIRMARY, LEEDS.

CHRISTMAS at the General Infirmary at Leeds has been a very happy time, and more so than has been possible for several years past, though in many respects a complete return to pre-war arrangements was not possible. The residents and nurses found it impossible, under the present stress of work, to carry out the entertainments which, prior to the war, were carried out in the large waiting-hall. The festivities commenced on Christmas Eve by the singing of carols by the boys of a local church choir, the boys afterwards being entertained to tea in one of the wards. This added to the enjoyment of the patients as much as to the singers themselves. Christmas fare in the form of turkeys, geese, roast sirloins of beef, Christmas pudding, fruit, sweets, and crackers were enjoyed throughout all the wards, the carving being carried out by residents suitably attired for the Christmas season. Special Christmas provisions were provided by special donations from friends of the institution. The domestic staff and porters joined in a suitable Christmas dinner, and afterwards paraded the

**Christmas in the Hospitals**—(*continued*).

wards in costumes and regaled the patients with carols and songs. A special dinner was provided for the nurses on Christmas evening, the carving again being done by the residents. On Boxing Day evening the porters and members of the domestic staff, to the number of 150, were entertained to a dance in the large Board Room. These entertainments were followed on subsequent days by a dance given to the nurses by the medical students, later by a dance given by the infirmary authorities to the nurses, residents, and senior students, and on other days dinner parties to various sections of the staff.

The general opinion expressed by patients and staff was that the Christmas festivities have passed off in an extremely happy manner, with the full vigour and pleasantness of the long past pre-war days.

### LEICESTER ROYAL INFIRMARY.

If the arrangements for the celebration of a real Peace Christmas entailed hard work and careful thought on a staff whose time never runs to waste, what mattered it when everything was so successful? The spirit of Christmas was evident, and the testimony of visitors and patients such as to give the greatest possible encouragement. It was precisely because the nursing staff were determined that their spare time should be used for the entertainment of their patients that the proceedings went through without a hitch, and without depending in the least on professional help. All the arrangements were supervised by Miss Vincent, the matron, with the assistance of Miss Marriott, the assistant matron, and the staff.

On Christmas morning each patient awoke to find himself the recipient of a useful gift; each adult male patient was also supplied with an ounce of tobacco or a supply of cigarettes. Although patients were early awake it was not too early for excited and eager recipients; one old Daddy, whose solemn face showed that he thought he was speaking truth, said, "He never had a wink of sleep all night," although "he could not think how the night nurse came to fill his stocking and place it on his bed without his seeing her and remembering." Christmas dinner consisted of roast beef, plum pudding, custard, bread and cheese, sweets, and fruit, and bon bons, while each adult patient was allowed bottled beer if he so desired. At 4 o'clock patients were allowed to invite a friend to tea, supplied by the institution.

At 5.30 Sister Marriott and her Musical Troupe of Nurses, who last year added largely to the gaiety of the festivities, presented an artistically arranged Gipsy Encampment of the George Borrow type, with real grass and big logs, and a

Photo.] The Children's Ward. [Dr. Gladstone.

caravan labelled "Not to let," displaying the product of gipsy skill and traditional characteristics in the shape of a poached duck and snared rabbit. The dresses were made by the nurses themselves, and with their gay colours, lavish trimmings, and the resplendent hat feathers, made the scene as realistic and gipsy-like as can be imagined. The gipsies were seated round camp fires (electric), some at play or telling fortunes with cards, others doing the domiciliary duties, washing, and preparing the meals. A moment or two's inactivity and the troupe rose to the centre of the ward to sing to action various songs and give a duologue, finishing up with a dance and the National Anthem. An excellent entertainment, artistically conceived and splendidly arranged, acted with real interest and general enthusiasm. Small wonder that the troupe had to repeat their performance on three evenings during the week in various wards, to the great delight of the patients and visitors.

On Boxing Day some sixty of the nurses, led by Miss Vincent, perambulated the wards at early evening, all lights extinguished except those from candles carried by the nurses, while they sang in every war familiar Christmas carols, to the great joy of the patients. In the Children's Hospital each of the three wards was provided with a Christmas-tree, and by the kindness of Mrs. Forester, who has on

Photo.] The Gipsy Caravan Troupe. [F. Lumbers, Leicester.

**Christmas in the Hospitals**—(*continued*).

previous occasions entertained the children, a Punch and Judy Show was arranged for, and gave delight to the little ones.

The illustrations accompanying this article are the work of Dr. Gladstone, the house physician.

### ROYAL VICTORIA INFIRMARY, NEWCASTLE-UPON-TYNE.

CHRISTMAS at the Royal Victoria Infirmary was celebrated on the old-fashioned plan. There were no innovations. Christmas is one of those institutions best left untampered with. Nurses singing carols in the wards ushered it in. There was a service in the infirmary chapel at 10.30 in the morning. Father Christmas (one of the resident doctors) distributed presents to every patient in the infirmary. Dinners in the wards were at 12 o'clock—turkey, plum pudding, and fruit. Afterwards various concert parties gave entertainments in the wards. All was over at 8 o'clock. The wards were prettily and inexpensively decorated. There was no over-decoration. The turkeys were admirably cooked, and the plum pudding had a pre-war savour. During the last four years gaiety at Christmas-time has been a little forced. This year there was a happier spirit. Everyone really enjoyed himself. There were, of course, the Christmas-trees (four) for the children laden with presents. There were also the resident doctors, who in costume gave a really first-rate performance. This year they christened themselves "The Liqueurs." Christmas Day is one which we all like to spend in the bosoms of our families. Yet during the course of the day every member of the honorary staff found an opportunity to come; often their wives and children came with them. Many of them helped to carve the turkeys in the wards. The hospital was also glad to welcome the Chief Magistrate of the City, the Lord Mayor, who, accompanied by the Lady Mayoress, went round the wards. The Lord Mayor took the opportunity of speaking to those who had been severely injured in a large fire in the City that took place a few days before Christmas.

### GENERAL HOSPITAL, NOTTINGHAM.

To many it may seem strange, but nurses know, nevertheless, it is true, that of all places a hospital is one of the best to spend Christmas in.

Festivities started on Christmas Eve at the Nottingham General Hospital, when, after supper, the nurses sang carols in the wards, much to the enjoyment of the patients. The children's ward, representing spring, was particularly effective, and the heavily-laden Christmas-tree gave unbounded joy to the youngsters. At twelve o'clock the patients' dinners were served, consisting of turkey, greens and potatoes, Christmas pudding, mince-pies and jellies, a glass of ale being allowed to all those patients who were able to take it. The turkeys were carved in the different wards by Mr. Acton, chairman of the Board, and the honorary medical staff, the resident medical officers acting as waiters. In the evening impromptu concerts were given in the wards. On December 26 the Christmas-tree was dismantled, Mr. Neil, honorary assistant surgeon, acting as Father Christmas. In the evening the nursing staff kept their Christmas, the Nurses' Home being decorated with flowers and shaded lights. Dancing and games finished the evening.

On Sunday afternoon, December 28, a service was held in huts usually occupied by pensioners (who had been allowed Christmas leave), when a rendering of "The Messiah" was given by the chorus and orchestra of the Sacred Harmonia Society, under the direction of Mr. Allen Gill, F.R.A.M., who came down specially from London to conduct. This performance was greatly appreciated by everyone, and many thanks are due to the artistes who so willingly gave their services.

On January 2 the nursing staff gave an entertainment to the patients and domestic staff, which was repeated the following evening to the members of the Board and visitors.

### GENERAL HOSPITAL, NORTHAMPTON.

CHRISTMAS was a very happy time at the Northampton General Hospital, for both patients and nurses. Festivities commenced on Christmas Eve, when sisters and nurses gathered in the main corridor to sing carols; each one carried a coloured lantern, and all the wards were visited. The matron and friends watched the procession from various points.

Christmas Day was excellent. The turkeys and plum pudding, together with other fare, were much appreciated by the patients. During the afternoon carols were again sung by choristers in the wards, and after tea patients were entertained in the various wards by the nurses.

On Boxing Day Santa Claus distributed presents from the tree to the children, and it was a pretty sight to see them, all dressed in red and white, eagerly waiting for their gift. That evening, matron entertained the sisters to dinner, and afterwards was good enough to take as many as could be spared from the wards to the theatre, which concluded a most delightful evening.

The following day outside friends gave a tea to all patients and nurses in the male wards, promising one to the others for Monday. Finally a grand concert was given in the accident ward, and a large gathering of patients and nurses from other wards enjoyed an excellent programme.

### ROYAL BERKSHIRE HOSPITAL, READING.

GREAT were the preparations made at the Royal Berkshire Hospital for the Christmas festivities this year. Late on Christmas Eve all was in readiness. Carefully-chosen parcels had been made for each patient; quietness reigned throughout the hospital, yet there was a feeling of expectation among those who watched the sleepers. When morning came great pleasure was shown at the useful and pretty presents. In the children's ward a large Christmas-tree stood laden with toys, and from each cot hung a bulky "stocking" filled with all that a child's heart could wish for. When the children awoke their delight was unbounded. The wards were soon in order, each having been decorated with much taste and wonderful variety, reflecting great credit on the sisters and nurses. The nurses spent a short time visiting the different wards, admiring the decorations, each returning to her own ward convinced that it was the best. The night nurses got up for tea, and joined in the singing of carols afterwards.

The nursing staff had dinner in two parties. The dining-room was effectively decorated, and the tables looked charming. The Matron presided at both dinners. The turkeys, Christmas puddings, and all the other good things were enjoyed by all. In the midst of fruit, crackers, and fun a member of the medical staff, who is the Mayor and, incidentally, one of the nurses' best friends, paid a visit, joining in the merriment and proposing the toast to the King. This was followed by others, with a special one and hearty cheers for the Matron, who had been so unsparing in the giving of her time and thought for the happiness of all under her care. At the conclusion of the dinner a bran-tub caused much fun, and very pretty were the presents each drew forth.

Concerts were given in the wards on Friday and Saturday. All the patients who could be moved were brought from the wards, and their hearty cheering showed how much they enjoyed the efforts to entertain them. One concert, given by some of the sisters and nurses, displayed much talent. Many of the doctors and members of the Committee also came to the concerts. On Monday the Mayor and Mayoress visited the wards, having a cheery word for all. An entertainment followed, with many amusing songs, which specially delighted the small boys present.

On Tuesday the Board of Management was "At Home," tea being served in the waiting hall; there was a large

**Christmas in the Hospitals**—(*continued*).

attendance, and the visitors afterwards went to the different wards to speak to the patients and admire the decorations. The guests then repaired to the children's ward, where good old Father Christmas paid a visit to the litle ones and distributed the toys from the Christmas-tree. Only those who have witnessed such an afternoon can fully appreciate the joy it gives.

### SALISBURY INFIRMARY.

THE keeping of Christmas at Salisbury Infirmary gave pleasure to patients and staff alike. For a week beforehand patients were mysteriously busy over the decorations, and secrets as to colour and design were carefully kept. In all cases the results were dainty and effective, and of an entirely different character to those which have been popular during the years of war. Lovely holly and evergreens were freely used. In one ward a floral scheme of purple and white clematis was much admired, the flowers having been cleverly made and arranged by the men patients. A butterfly scheme and a representation of winter were also greatly admired.

At 9 A.M. on Christmas Day Father Christmas (personated ably by the dispenser), accompanied by a band of Girl Guides with their captain, made a tour of the wards, and distributed parcels to every patient. These parcels contained very useful and pretty gifts, which had been individually chosen for them at the request of the friends who had sent money for the gifts.

At noon special dinners gave great satisfaction. In the men's wards turkey was provided. The women and children were given chickens. These had been grown on the hospital poultry run, and were of fine quality. Plum pudding and jelly, with ale or lemonade, made up the meal. The visiting staff and the house surgeons carved in the different wards, much to the satisfaction of the sisters.

Radnor Ward, Salisbury Infirmary.

The time-honoured custom of allowing each patient to invite two friends to tea had been followed, and about 3 P.M. numerous visitors began to arrive, many of them having walked long distances in the rain, which fell heavily during most of the day. The ward teas were provided by the lady ward visitors who were present and presiding at the tables. Many visitors, among them the Chairman, the Mayor, members of the committee and visiting staff, with their wives and children, came during the afternoon, taking great interest in the patients and staff. After tea a choir, composed of members of the nursing and household staffs, sang carols in all the wards. The singing was excellent, and was much enjoyed.

On Tuesday an entertainment was given in the large Board-room by the nurses. Two sketches, "Snowed up with the Duchess" and "Mechanical Jane," were well acted, and caused much amusement. Country dances and singing filled in the programme. The dancers wore most dainty costumes, cleverly made by themselves. The "Pensioners," who all had leave to go home for Christmas, had returned in time for the entertainment. They received useful gifts on New Year's morning, had special fare, and a ward tea provided for themselves and their friends on that day. This was by the kind arrangement of their ward visitor. Although the thought of keeping Christmas in these expensive days gave some cause for anxiety beforehand, those anxieties were entirely removed by the very kind response given to the special appeal. This special appeal is sent out annually in the joint names of the matron and the chaplain. Gifts in money and kind were so generously sent that every item of expenditure will be covered, and no expense for any of the festivities will come upon the general fund.

### GENERAL INFIRMARY, STOCKPORT.

THE wards and corridors wore a festive air, having been artistically decorated with lamp shades and evergreens; each ward and corridor had its own particular colour scheme. A striking feature of the decorations was the corridor from the main building to the out-patients', which was gay with artificial rose-trees in "bloom," and was appropriately described as the "Artists' Walk." An illuminated Christmas-tree proved a great attraction in the children's ward, and there was an exceptional cheerfulness, brightness, and charm about the whole of the wards. Every patient in the hospital hung up a stocking or a pillow-slip. In the children's ward the patients were unduly wakeful on Christmas Eve, which made the task of filling stockings, or rather pillow-slips, a difficult one, until Night Sister conceived the happy idea of "garbing" herself as Father Christmas for the purpose, and this was successful in allaying any doubts which they might have as to Father Christmas being real.

On Christmas morning the nurses commenced the day by singing carols in the wards, and later in the morning an aged friend of the hospital, dressed up as Father Christmas, distributed a useful present to every patient. During this proceeding the Maia Choir, consisting of sixty voices, rendered Christmas carols. This choir has visited the hospital on Christmas morning for seventeen years, and it was remarked that they had never sung better. The Borough Band and Industrial School Boys' Band also played selections of music during the morning. The patients dined at 12 noon off roast turkey or chicken, plum pudding, and dessert. The carving was done by friends of the hospital. At four o'clock the patients had tea, when various delicacies were provided. Impromptu concerts were the order of the evening.

On Boxing Day the nurses presented a sketch, entitled "Blatherwick's Diplomacy," in the children's ward, where a stage had been erected, and this was thoroughly enjoyed and appreciated by every patient who was able to be moved from their wards. On Saturday a dance entertainment was given by an old patient, a very clever and versatile girl dancer of ten years of age, when all patients who were well enough to be moved enjoyed the performance. At 8 P.M. on Saturday the nursing staff had their annual dinner, followed by a whist drive. The annual fancy dress dance will be held early in the New Year.

The Christmas-tree distribution is the event which takes pride of place in the festivities, and this was held on Monday, December 29. About 150 visitors were entertained to tea, and the patients also had a special tea provided for this day. Words fail to describe the happiness reflected on the children's faces as the hour approached for which

**Christmas in the Hospitals**—(*continued*).

they had waited for several days, and as the visitors moved from bed to bed speaking kindly words to the youngsters there was no mistaking what was foremost in the minds of the occupants.

On Tuesday a concert party, arranged by a grateful patient who had left the hospital, gave a very enjoyable performance, which was much appreciated by all who were able to attend.

### THE ROYAL ALBERT EDWARD INFIRMARY, WIGAN.

The infirmary interior was gaily decorated with an abundance of flags, streamers, Chinese lanterns, fairy lights, holly, etc., a most picturesque effect being produced. Christmas was heralded by the singing of carols in the wards by certain of the nursing staff, and their excellent efforts did much to cheer the bedridden sufferers. The Chairman (Mr. Wm. Johnson) and the matron (Miss M. K. Coggins) visited the patients, all of whom received Christmas fare to such extent as they were able to partake of. This consisted of turkey, plum pudding, a variety of confections, etc. The adult patients received appropriate gifts, and each child a Christmas stocking.

On the evening of December 27 members of the Board of Management and of the honorary staffs and their friends assembled in the "Upper Johnson" ward to witness the dismantling of the in-patients' Christmas-tree. Dr. James Brooks, the senior house surgeon, excellently filling the rôle of Father Christmas. The children and members of the various staffs were the happy recipients of presents of an infinitely varied kind, not a few of which were such as to make the onlookers laugh with much heartiness. Amid no little amusement Mr. Hedley Lucas (the General Superintendent and Secretary) received for the infirmary a cheque for £1,000,000, the gift of "Father Christmas," but though the benefactor's funds are unlimited the irony is that his generosity is not bestowed through the medium of any bank, and the cheque consequently remains "unhonoured"!

Prior to the foregoing proceedings Mr. and Mrs. John Prestt and family visited the "Lower Johnson" ward for the purpose of viewing the two cots which they have so kindly endowed in memory of their son and brother and of a friend, whereby the infirmary funds have benefited to the most welcome extent of £1,000. They were accompanied by two members of the Board and the General Superintendent and Secretary, and in an unostentatious manner a ceremony of deep and abiding significance was witnessed.

On December 29, thanks to the kindness and the further untiring efforts of the nursing staff, an excellent concert was given in the large out-patients' hall, some 300 persons being present. The concert was a great success, and the excellent sum of £21 10s. 2d. has been realised for the hospital. In response to many requests the entertainment will be repeated.

On December 30 some sixty out-patient children were entertained in the out-patients' hall. They did full justice to the splendid Christmas tea which was provided for them upon tastefully decorated tables, everything possible being done to promote their happiness. When the screened Christmas-tree was revealed there were many exclamations of joy from the little ones. From the tree, gleaming with fairy lights, wondrous in colour, and laden with such gifts as delight the hearts of children, "Father Christmas," in the person of the senior house surgeon, distributed two presents to each child guest. At the close an added pleasurable touch was given to the proceedings by the presentation to Miss Coggins (the matron) of a lovely bouquet of lilies of the valley, the kind and acceptable gift of an out-patient.

The nursing staff had also a fancy-dress ball and whist-drive, excellent and useful prizes, including a silver-top cut-glass scent bottle and a silver manicure set, being very kindly provided by Mr. William Johnson, the chairman.

### ROYAL HAMPSHIRE COUNTY HOSPITAL, WINCHESTER.

Although, with the exception of a few pensioners, the Service patients have gone, the wards of the Royal Hampshire County Hospital are fully occupied, and the patients are of all ages, from little tots to persons advanced in years. The sisters and nurses made themselves responsible for the decoration of the wards, and they had the cheerful co-operation of such of the patients as were able to render assistance. The decorations throughout were floral. On the ground-level flowers of the earlier season of the year were entwined with evergreens. In the Bartlett Ward there was a charming effect from the introduction of clematis of different colours, a clematis-covered archway at the far end being especially noticeable. A big bough of laburnum in the central window was also a striking feature. Owing to structural work in progress it has been necessary to use the Heathcote Ward on the same floor as a temporary dining-hall for nurses, and therefore the decorations here were not elaborate. But it was difficult at first sight to believe the tulips embedded in moss in the window-sills were merely artificial. It should be mentioned that the whole of the (paper) flowers were made by the nursing staff, with, in one or two instances, skilful assistance from patients. The Nightingale Ward was chiefly distinguished by poinsettias. In the Victoria Ward the scheme carried out was that of "Dawn in a Rose Garden," and here the deft fingers of one of the girl patients conspicuously assisted in the making of the dainty roses that clambered over trellis-work, and formed a pergola at the far end. In the children's ward Fairyland appealed to the fancy of the little ones; there was a fairy grotto with coloured lamps; there were dolls and toys among the spring flowers; in the windows fairies shaded the electric light; and, to crown all, there was a huge Christmas-tree laden with goodies and presents of all kinds. In the younger children's room adjoining there was a pretty rose-coloured trellis. Throughout the building evergreen was in abundance, and the electric light fittings were covered with shades in cheerful colours.

At 9 o'clock on Christmas morning an airship (the construction of which was chiefly due to Mr. J. McMurray Cole, the house surgeon) visited the whole of the wards, and distributed acceptable gifts to all the patients—grown-ups and juveniles. Turkeys, Christmas puddings, etc., were in the menu for dinner, and there was a special tea. At 5 o'clock the nurses and others visited the wards and sang carols. At half-past seven the sisters and nurses had their Christmas dinner, and there was afterwards a rehearsal of the programme arranged for the entertainment the following Monday evening. The Christmas-tree of the children's ward was the event of Boxing Day. The fine tree had been given by Dr. Sedgwick, of Bereweeke Road, and presents galore were sent in for the tree by friends. It was charmingly lighted with coloured electric lamps (by Messrs. Dicks, Ltd.). Not only the actual occupants of the ward but the whole of the children who had been patients during the past year were invited to a hugely enjoyed tea before the tree. Father Christmas (Dr. Cole) arrived in his airship with two little fairy attendants (Miss Pardoe and Miss Campbell), a gnome (Miss Betty Briggs), and one of the child patients in the dress of an Austrian peasant. They set to work stripping the tree; the Matron, Miss Carpenter Turner, and others helped, and soon the gifts passed into the ownership of many.

On Christmas Day there was a celebration of Holy Communion in the chapel at 6.30 and 7.30 in the morning, Divine service following later on. At 5 o'clock in the afternoon the nurses started singing in the vaious wards. The services were conducted by the Rev. B. T. Pitts. The collections during the day were for the Waifs and Strays Society. On Sunday evening there was a special carol service at 5 o'clock. The nursing staff was augmented by singers from Winchester College, Holy Trinity, and St. Thomas's choirs (who kindly gave their services). Being Holy Innocents' Day the service was specially meant for and attended by young people. The prayers were taken by the Rev. B. T. Pitts, and the preacher was the Rev. C. R. Hollis (curate of Hursley).

## Figures and Tables

**Figure f1:**
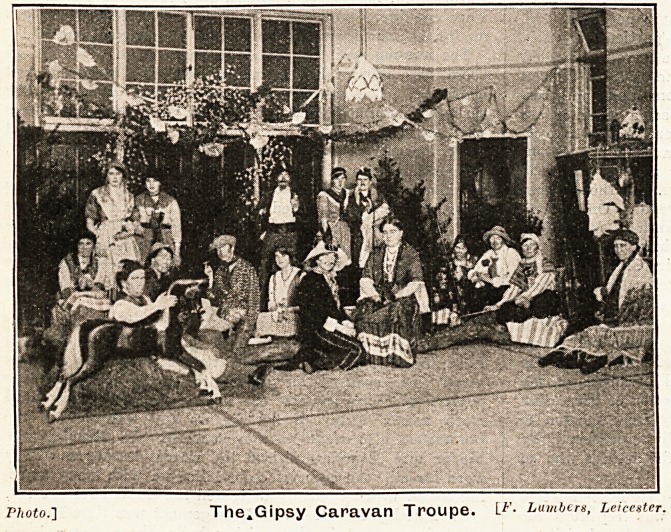


**Figure f2:**
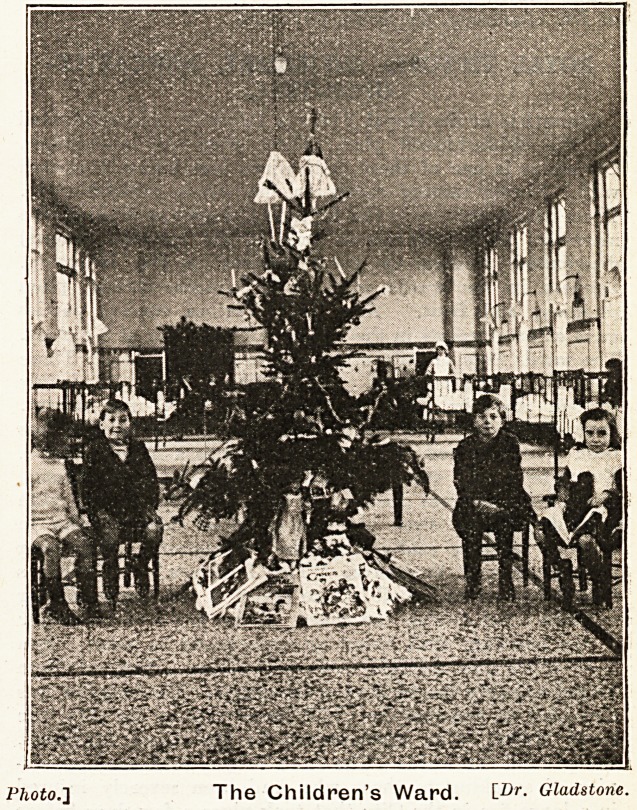


**Figure f3:**